# Supplementary material for: Insights into the client protein release mechanism of the ATP-independent chaperone Spy
Source: Nat Commun. 2022 May 20;13:2818. doi: 10.1038/s41467-022-30499-x (PMC9122904; doi:10.1038/s41467-022-30499-x)
Supplement: Supplementary file 1 — Supplementary Information [file 41467_2022_30499_MOESM1_ESM.pdf]

**Supplementary Information**

# Insights into the client protein release mechanism of the ATP-independent chaperone Spy

Wei He *et al*.

**Supplementary Fig. 1**

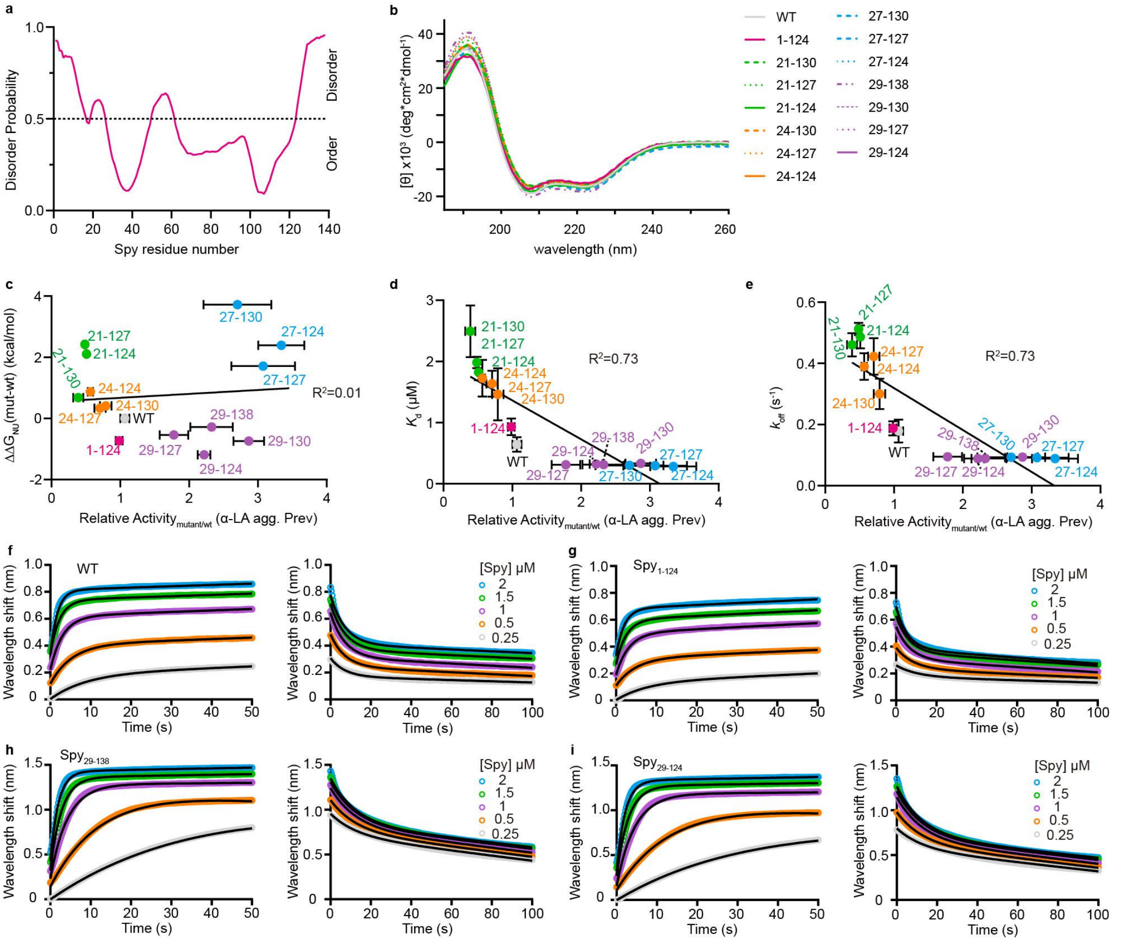


**Supplementary Fig. 1 Characterization of Spy wild type and termini-truncated Spy variants.** (a) Prediction of disordered regions in Spy using the PrDOS server[1]. (b) Circular dichroism spectra of Spy wild type and termini-truncated Spy variants. (c) Relationship between the chaperone activities and thermodynamic stabilities of termini-truncated Spy variants (mean ± SD, n = 3 independent experiments). (d-e) Correlations between the chaperone activities of termini-truncated Spy variants and their dissociation constants $K_d$ (d), or dissociation rate constants $k_{off}$ (e) for the model client protein $Im7_{AAW}$ (mean ± SD, n = 3 independent experiments). For (c-e), the chaperone activities of Spy wild type and termini-truncated Spy variants are their abilities to prevent the aggregation of DTT-reduced α-LA. Abbreviation α-LA agg. Prev means α-LA aggregation Prevention. (f-i) Representative kinetic curves of the association and dissociation of

various concentrations of Spy wild type (f), $Spy_{1-124}$ (g), $Spy_{29-138}$ (h), and $Spy_{29-124}$ (i) with biotin-labeled $Im7_{AAW}$. Source data are provided as a Source Data file.

**Supplementary Fig. 2**

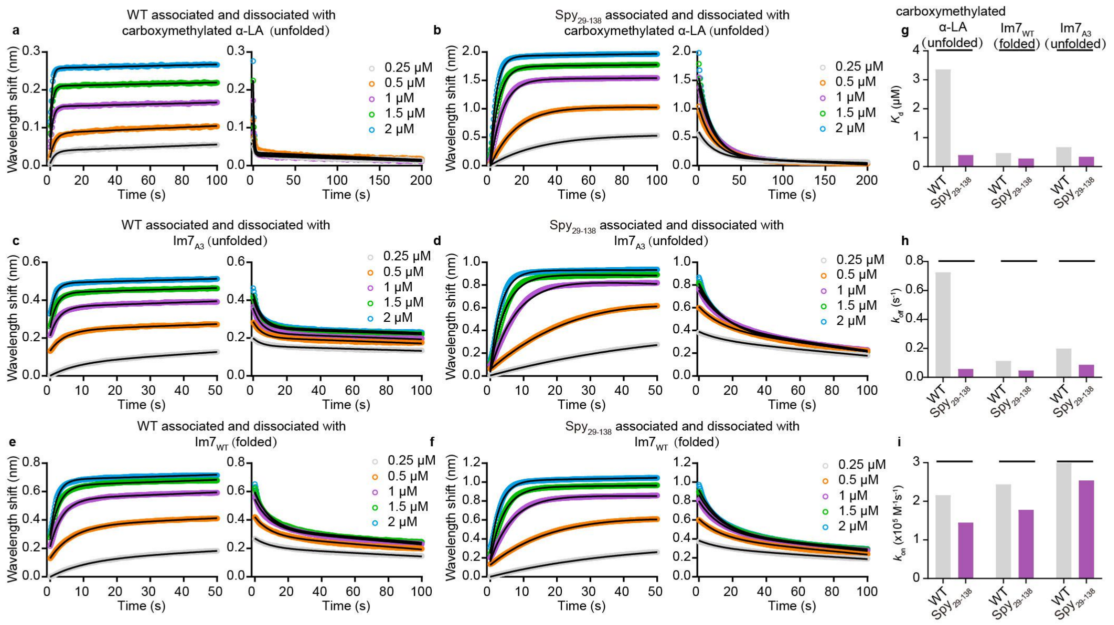


**Supplementary Fig. 2 The N-terminus of Spy facilitates client release.** (a-f) Association and dissociation kinetic curves of various concentrations of Spy wild type and Spy$_{29\text{-}138}$ towards carboxymethylated α-LA (a-b), or Im7A$_3$ (c-d), or Im7$_{WT}$ (e-f). Carboxymethylated α-LA and Im7A$_3$ are model chaperone clients trapped in unfolded states, while Im7$_{WT}$ is folded. Kinetics curves were determined by bio-layer interferometry using the same concentration gradient of Spy wild type and Spy$_{29\text{-}138}$. (g-i) The dissociation constants $K_d$ (g), dissociation rate constants $k_{off}$ (h), and association rate constants $k_{on}$ (i) of Spy wild type and Spy$_{29\text{-}138}$ towards carboxymethylated α-LA, Im7A$_3$, and Im7$_{WT}$. Source data are provided as a Source Data file.

Supplementary Fig. 3

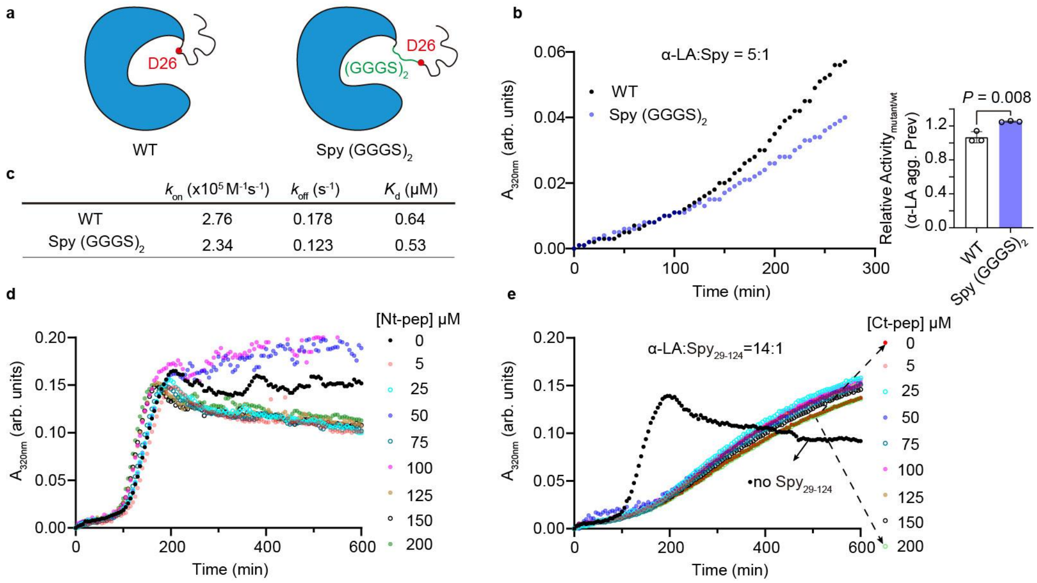


**Supplementary Fig. 3 The disordered N terminus of Spy affects its chaperone function.** (a) Schematic representation for the positions of the N terminus in Spy wild type and the Spy variant with a (GGGS)$_2$ linker inserted between D26 and M27. The disordered N terminus is indicated by a black line, while the (GGGS)$_2$ linker is indicated by a short green line. The D26 residue is indicated by a red dot. (b) Aggregation curves of 50 μM DTT-reduced α-LA in the presence of 10 μM of Spy wild type and the Spy (GGGS)$_2$ variant. Quantification of the anti-aggregation activities of Spy wild type and Spy (GGGS)$_2$ is shown in the bar graph to the right (mean ± SD, n = 3 independent experiments, individual data points are shown; unpaired two-tailed student t-test). Abbreviation α-LA agg. Prev means α-LA aggregation Prevention. Statistical analysis was performed with Graphpad Prism 9.1.0. (c) Kinetic parameters of Spy wild type and Spy (GGGS)$_2$ towards Im7$_{AAW}$. (d) Aggregation curves of 50 μM DTT-reduced α-LA in the presence of various concentrations of Nt-pep. (e) The anti-aggregation activity of Spy$_{29-124}$ in the absence or presence of various concentrations of Ct-pep. Representative curves from three independent measurements are shown in (b), (d), and (e). Source data are provided as a Source Data file.

Supplementary Fig. 4

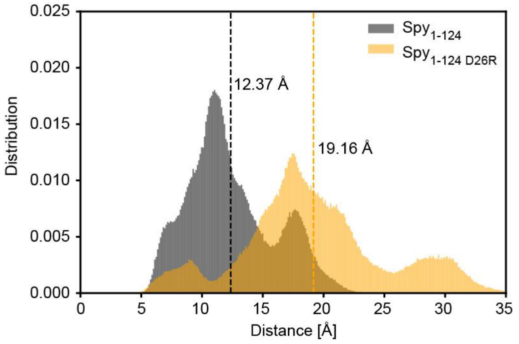


**Supplementary Fig. 4 Distributions of the distance between residue 26 and the cavity of Spy as observed in the MD simulations.** The normalized distributions for the $Spy_{1\text{-}124}$ and $Spy_{1\text{-}124\ D26R}$ are shown in black and orange, respectively, with average distances indicated as dashed lines in corresponding colors. The intramolecular distance was calculated using the geometry center of all atoms in D26/R26 and the geometry center of the cavity region (residues 29-124) for each monomer, after combining four MD trajectories with different initial conformations.

Source data are provided as a Source Data file.

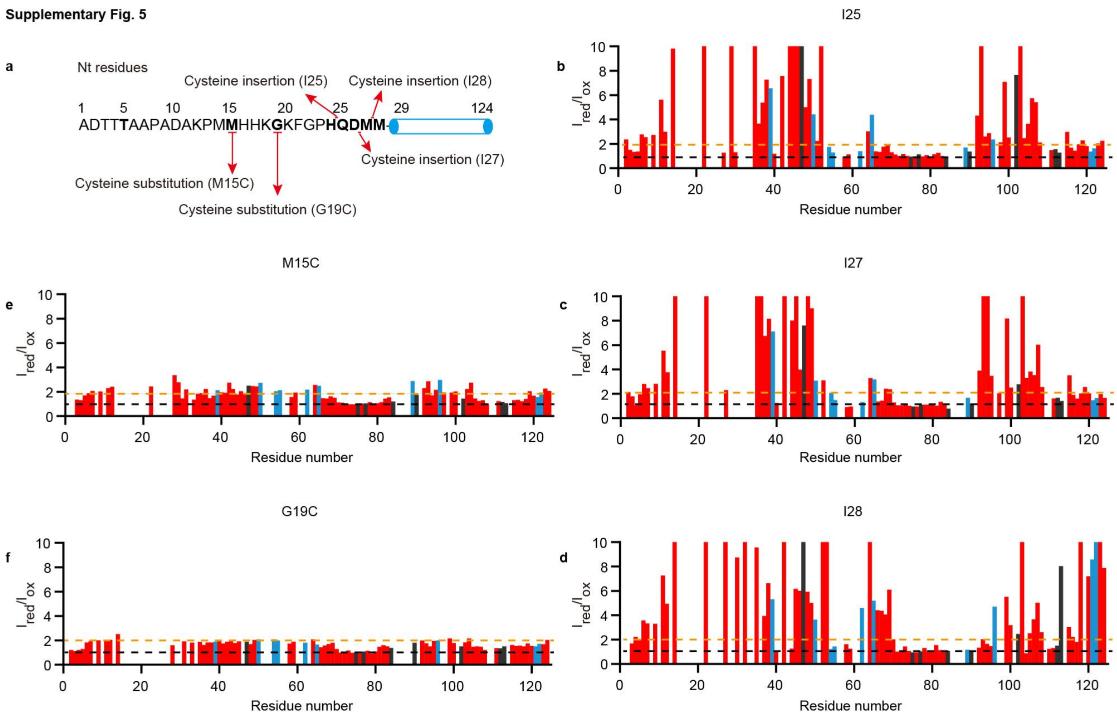


**Supplementary Fig. 5 MTSL spin labels attached to the cysteine residues inserted near D26 induce strong intramolecular PRE effects.** (a) Illustrations of Spy variants used to perform intramolecular PRE experiments. (b-f) Intramolecular PRE effect of the spin label on $^{15}$N-labeled Spy$_{1\text{-}124}$ variants in which the paramagnetic spin label (MTSL) is attached to a cysteine inserted before residue Q25 (I25), or before M27 (I27), or before M28 (I28), or to a cysteine substitution at M15 (M15C) or G19 (G19C). The PRE effects were presented by the ratios of peak intensities before and after the reduction of the spin label ($I_{red}/I_{ox}$). PRE intensity ratios of 1 and 2 are indicated by black and orange dashed lines, respectively. Positively charged residues with side chains pointing to the concave or convex surfaces of Spy are marked in light blue and black, respectively. Source data are provided as a Source Data file.

**Supplementary Fig. 6**

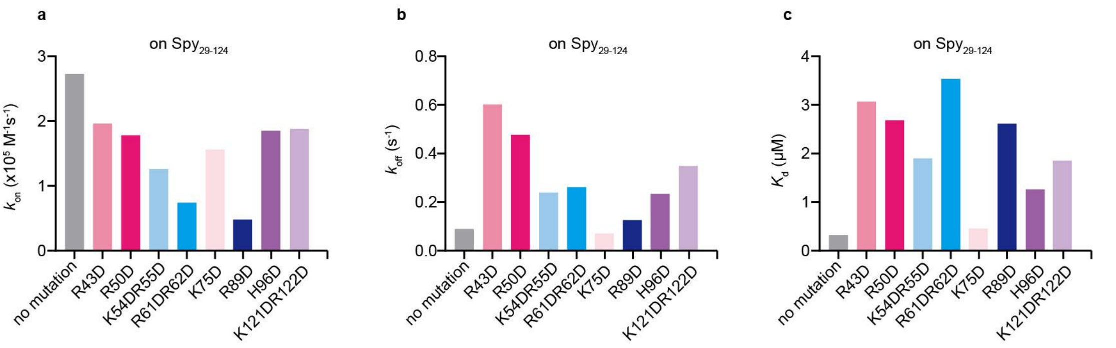


**Supplementary Fig. 6 Kinetic characterization of potential D26-interacting residues on Spy's concave surface.** Various mutations were constructed on $Spy_{29\text{-}124}$, and their association and dissociation kinetics for the $Im7_{AAW}$ client were monitored by bio-layer interferometry. Bar graphs show the association rate constants $k_{on}$ (a), dissociation rate constants $k_{off}$ (b), and dissociation constants $K_d$ (c) of these variants compared with $Spy_{29\text{-}124}$. In the absence of the N terminus, all of these aspartate variants including the K75D control decreased the $k_{on}$ rates (43% for K75D, 28-82 % for other aspartate variants), but only the ones with the aspartate substitution on the concave surface of Spy increased the $k_{off}$ rates, resulting in dramatic increases of the $K_d$.
Source data are provided as a Source Data file.

**Supplementary Fig. 7**

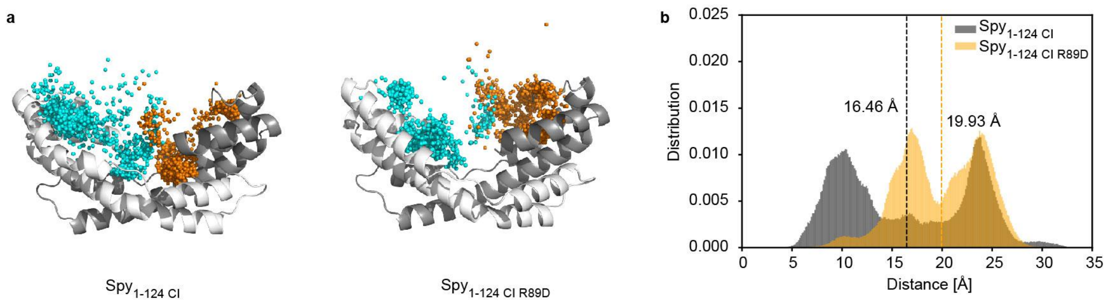


**Supplementary Fig. 7 Distributions of the distance between the MTSL label and the bottom of Spy cavity as observed in the MD simulations.** (a) Position of the MTSL spin label on the Spy cavity for MD simulations of Spy$_{1\text{-}124\ \mathrm{CI}}$ (left) and Spy$_{1\text{-}124\ \mathrm{CI\ R89D}}$ (right). The two monomers of Spy are shown as cartoons in white and gray, and the nitroxide oxygen (O1) atoms of spin labels from different monomers are represented as spheres in cyan and orange, respectively. The statistics was performed using 1000 MD frames uniformly resampled in the combined trajectory from four MD simulations with different initial conformations of N terminus. Compared to Spy$_{1\text{-}124\ \mathrm{CI}}$, the O1 atoms of Spy$_{1\text{-}124\ \mathrm{CI\ R89D}}$ evidently moved away from the bottom of the cavity. (b) The normalized distributions of the distance between the MTSL label and the bottom of Spy cavity for the Spy$_{1\text{-}124\ \mathrm{CI}}$ (black) and Spy$_{1\text{-}124\ \mathrm{CI\ R89D}}$ (orange), with average distances indicated as dashed lines in corresponding colors. The intramolecular distance was calculated using the O1 atom of MTSL and the geometry center of all atoms in R89/D89 for each monomer, after combining four MD trajectories with different initial conformations. Source data are provided as a Source Data file.

Supplementary Fig. 8

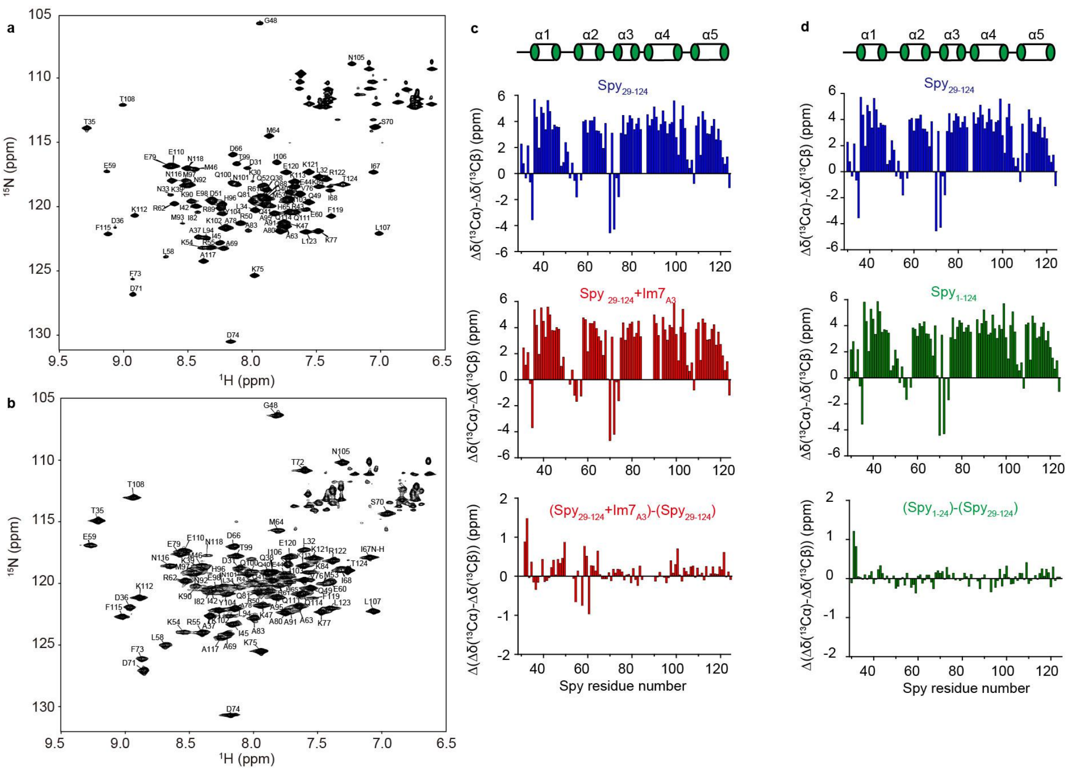


**Supplementary Fig. 8 Client binding or the presence of the N terminus do not extensively alter the secondary structure of Spy$_{29\text{-}124}$.** (a-b) The 2D [$^{15}$N,$^{1}$H]-TROSY spectrum of 400 μM [$^{2}$H,$^{13}$C, $^{15}$N]-labeled Spy$_{29\text{-}124}$ in the absence (a) or presence (b) of 400 μM Im7$_{A3}$. Sequence-specific resonance assignments of the backbone amide groups are labeled. (c) Secondary backbone $^{13}$C chemical shifts of Spy$_{29\text{-}124}$ (upper panel), Spy$_{29\text{-}124}$-Im7$_{A3}$ complex (middle panel), and their differences (lower panel) plotted against Spy residue number. (d) Secondary backbone $^{13}$C chemical shifts of Spy$_{29\text{-}124}$ (upper panel), Spy$_{1\text{-}124}$ (middle panel), and their differences (lower panel) plotted against Spy residue number. For (c-d), the secondary structures of Spy$_{29\text{-}124}$ are indicated on the top with cylinders denoting α-Helices. Source data are provided as a Source Data file.

**Supplementary Fig. 9**

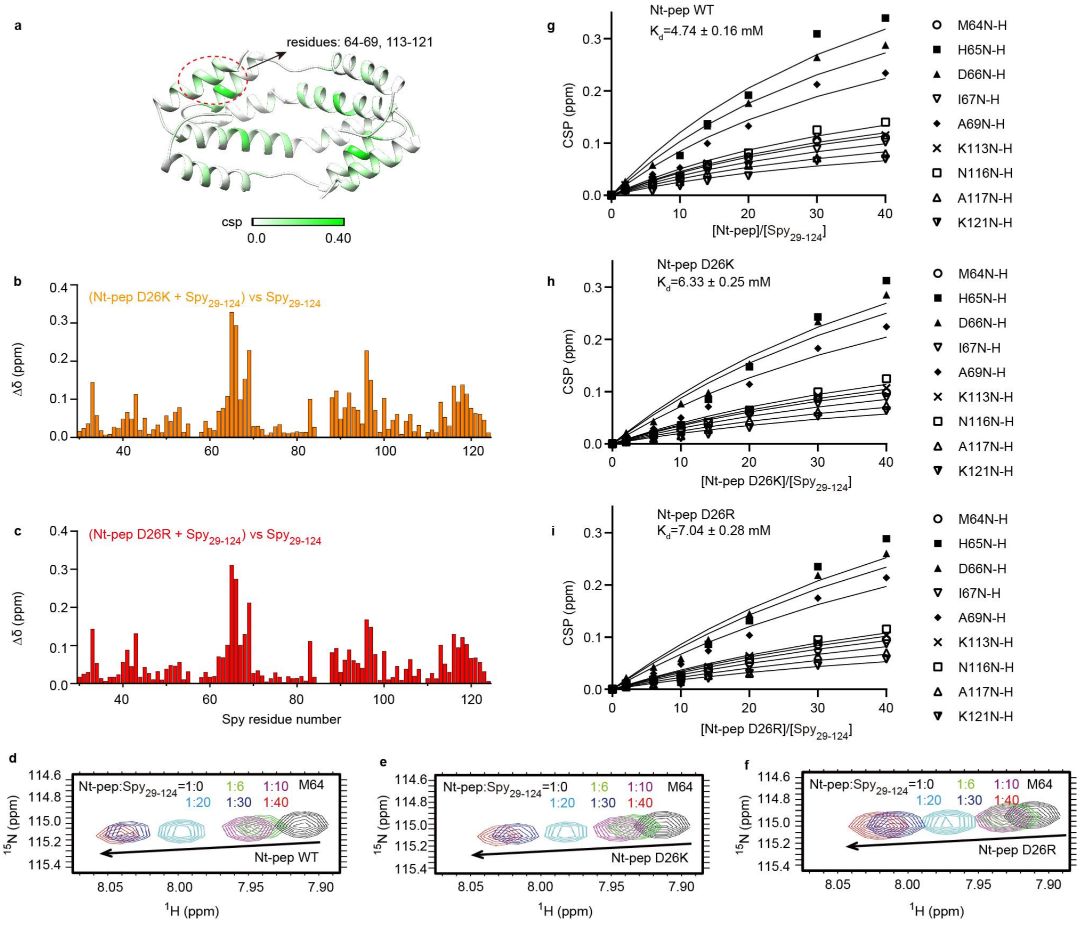


**Supplementary Fig. 9 The free Nt-pep has a weak affinity towards $Spy_{29-124}$.** (a) Display of the CSPs of 100 μM [$^{15}$N]-labeled $Spy_{29-124}$ upon binding with 4 mM unlabeled Nt-pep on the structure of $Spy_{29-124}$ (PDB: 3O39). A white-to-green scale was used based on CSPs ranging from 0 to 0.4. The region within the red dashed circle contains the residues whose CSPs were used to determine the dissociation constant ($K_d$) between Nt-pep and $Spy_{29-124}$ in the NMR titration experiments. (b-c) CSPs of the amide moieties of 100 μM [$^{15}$N]-labeled $Spy_{29-124}$ upon binding to 4 mM unlabeled Nt-pep D26K (b) or Nt-pep D26R (c) were determined and plotted against the residue number of $Spy_{29-124}$. (d-f) An overlay of the resonance peaks of residue M64 on $Spy_{29-124}$ upon titration with increasing concentrations of Nt-pep (d), Nt-pep D26K (e), or Nt-pep D26R (f), respectively. (g-i) Affinities of Nt-pep (g), Nt-pep D26K (h), and Nt-pep D26R (i) for $Spy_{29-124}$ determined by NMR titration experiments. The CSPs of eight residues located in the circled

region in (a) were used for $K_d$ calculations, including M64, H65, D66, I67, A69, K113, N116, A117, and K121. Black curves are global fittings to the data points. Note: Titration of Nt-pep to saturate $Spy_{29-124}$ was not feasible due to the solubility limitation of Nt-pep. Therefore, the $K_d$ value can only be considered as an estimate and the influence of non-specific binding between Nt-pep and $Spy_{29-124}$ cannot be completely excluded. Source data are provided as a Source Data file.

**Supplementary Fig. 10**

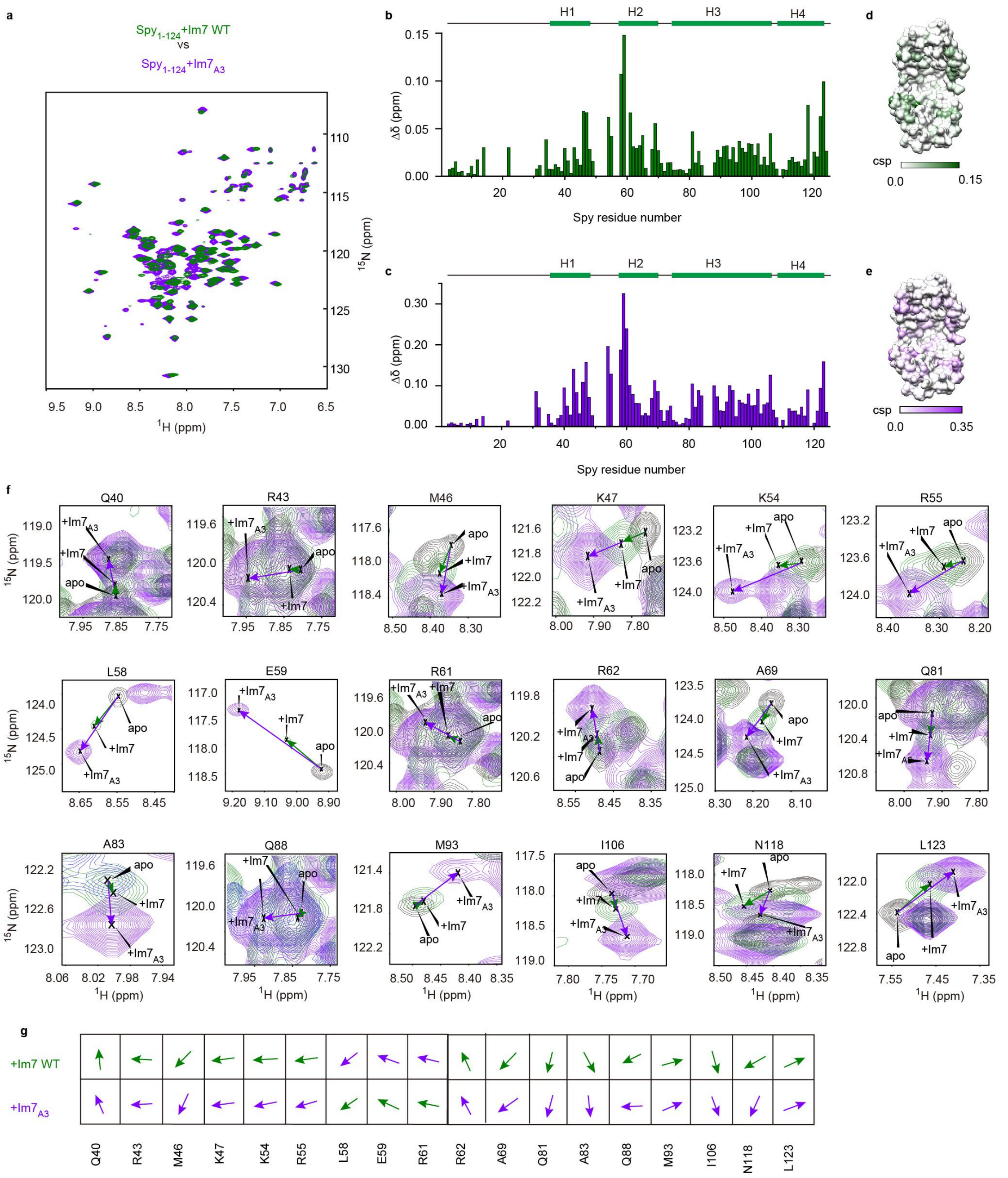


**Supplementary Fig. 10 Unfolded and folded Im7 variants have similar binding patterns on Spy.** (a) An overlay of the 2D [$^{15}$N,$^{1}$H]-TROSY spectra of 400 μM [$^{2}$H, $^{13}$C, $^{15}$N]-labeled $Spy_{1-124}$ upon binding with 400 μM unlabeled Im7 WT (green) or 400 μM unlabeled $Im7_{A3}$ (purple). (b-c) The CSPs of amide moieties of $Spy_{1-124}$ upon binding with Im7 WT (b) or $Im7_{A3}$ (c) were calculated and plotted against the residue number of $Spy_{1-124}$. The secondary structures of $Spy_{1-124}$ are indicated on the top with green bars denoting α-Helices. (d-e) Surface presentations of $Spy_{1-124}$

(PDB: 3O39), colored according to the CSP values corresponding to (b-c) with a two-color scale. Color intensities correlate with CSP values, with darker colors indicating larger values. (f) Resonance changes of residues with large CSPs upon binding with Im7 WT (green) or $Im7_{A3}$ (purple). Arrows indicate the direction of chemical shift changes upon client binding. (g) Summary of the direction of chemical shift changes of residues on $Spy_{1-124}$ upon binding with Im7 WT (green) or $Im7_{A3}$ (purple). Source data are provided as a Source Data file..

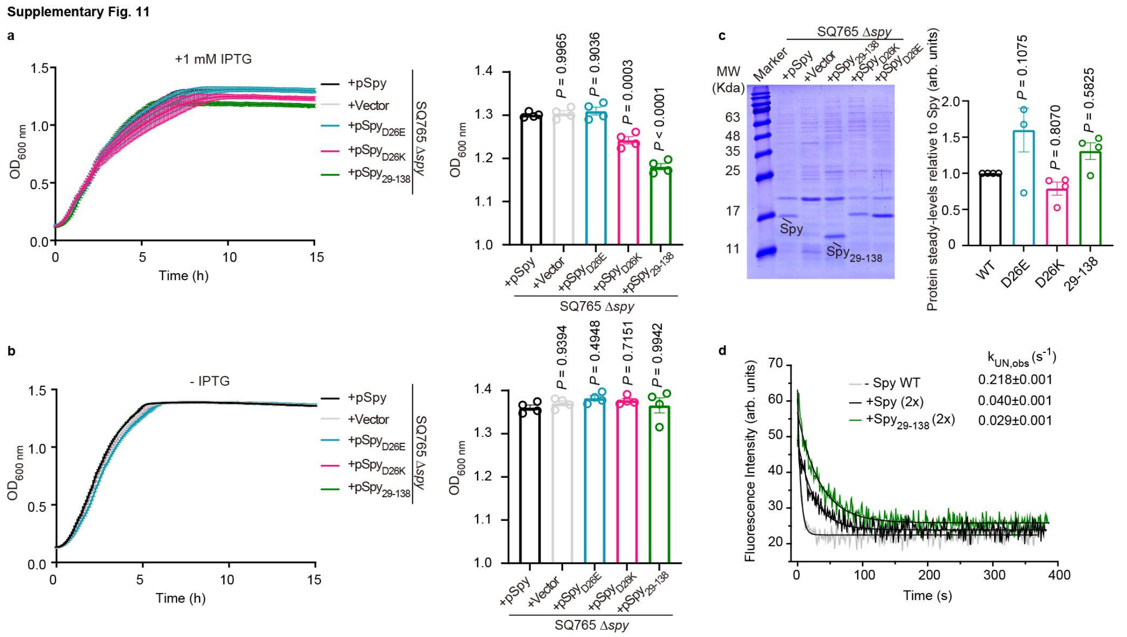


**Supplementary Fig. 11 Deleting the N terminus of Spy impedes client refolding and inhibits *E. coli* growth.** (a-b) Growth curves of *E. coli* strains carrying the expression plasmid of Spy wild type or $Spy_{D26E}$, $Spy_{D26K}$, or $Spy_{29\text{-}138}$, or the empty vector, with (a) or without (b) IPTG induction. Overnight cultures of different strains were diluted 100-fold into fresh LB media. After 2 h of growth, the cultures were treated with or without 1 mM IPTG for 2h and then with 1% butanol for another 1.5 h. Then, 0.1 OD cells from these cultures were inoculated into fresh LB media supplied with (a) or without (b) 1 mM IPTG, and cell growth was monitored. Comparisons of $OD_{600nm}$ values for various cultures at t = 12 h are shown to the right (mean ± SD, n = 4 biologically independent samples; individual data points are shown; one-way ANOVA with Tukey's multiple comparisons test). (c) For the IPTG-induced cultures in (a), samples were collected at t = 9 h, the periplasmic fractions of the cells were extracted and visualized on SDS-PAGE. Quantification of the band intensities was performed with Image J 1.52a. The band intensities corresponding to Spy wild type or Spy variants in arbitrary units (arb. units) are shown

to the right (mean ± SD, n = 4 biologically independent samples; individual data points are shown; one-way ANOVA with Tukey's multiple comparisons test). (d) Kinetic traces of urea-denatured α-LA (1 μM) refolded in the absence or presence of 2 μM Spy wild type or $Spy_{29\text{-}138}$, monitored by intrinsic tryptophan fluorescence. The observed refolding rates ($k_{UN,\ obs}$) were obtained by fitting the kinetic curves with a single exponential equation. Representative traces of two independent experiments are shown. Statistical analysis in (a) to (c) was performed with Graphpad Prism 9.1.0. Source data are provided as a Source Data file.

Supplementary Fig. 12

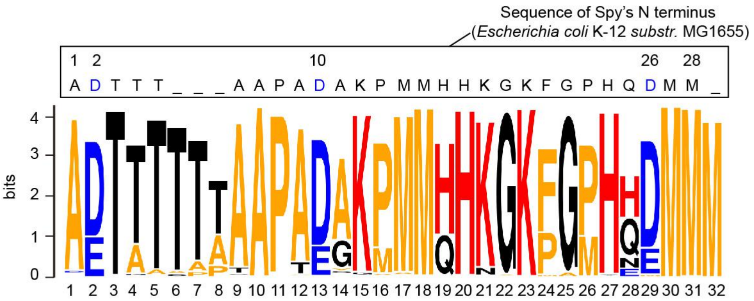


**Supplementary Fig. 12 Sequence conservation of the N termini of Spy homologs.** Sequences of the CpxP/Spy family were retrieved from the UniProt database. To separate the sequences of Spy orthologs from other homologous sequences, we built a phylogenetic tree using MegaX[2], and obtained 231 Spy sequences. After pairwise alignment of these sequences to the $Spy_{29-124}$ sequence from *Escherichia coli* K-12 *substr.* MG1655 and removal of the secretion signal sequences, we obtained 224 sequences corresponding to Spy's N termini from 54 species. After sequence alignment with MUSCLE[3], we created the WebLogo[4] of Spy N terminus. For comparison, the N terminal sequence of Spy in *Escherichia coli* K-12 *substr.* MG1655 is shown on top.

## Supplementary References


1. Ishida, T. & Kinoshita, K. PrDOS: prediction of disordered protein regions from amino acid sequence. *Nucleic Acids Res* **35**, W460-W464 (2007).
2. Kumar, S., Stecher, G., Li, M., Knyaz, C. & Tamura, K. MEGA X: Molecular Evolutionary Genetics Analysis across Computing Platforms. *Mol Biol Evol* **35**, 1547-1549 (2018).
3. Edgar, R.C. MUSCLE: multiple sequence alignment with high accuracy and high throughput. *Nucleic Acids Res* **32**, 1792-1797 (2004).
4. Crooks, G.E., Hon, G., Chandonia, J.M. & Brenner, S.E. WebLogo: A sequence logo generator. *Genome Res* **14**, 1188-1190 (2004).
