# Supplementary material for: Insights into the client protein release mechanism of the ATP-independent chaperone Spy
Source: Nat Commun. 2022 May 20;13:2818. doi: 10.1038/s41467-022-30499-x (PMC9122904; doi:10.1038/s41467-022-30499-x)
Supplement: Supplementary file 3 — Description of Additional Supplementary Files [file 41467_2022_30499_MOESM3_ESM.docx]

File name: Supplementary Movie 1

Description: 1 μs MD simulations of Spy wild type. The N and C termini of Spy are colored in red and blue, respectively. The D26 residues are displayed in stick formats with all atoms colored in dark red.

File name: Supplementary Movie 2

Description: 1 μs MD simulations of Spy_D26R_. The N and C termini of Spy are colored in red and blue, respectively. The R26 residues are displayed in stick formats with all atoms colored in dark red.

File name: Supplementary Data 1:

Description: Initial and final configurations of MD simulations.
