# Supplementary material for: Insights into the client protein release mechanism of the ATP-independent chaperone Spy
Source: Nat Commun. 2022 May 20;13:2818. doi: 10.1038/s41467-022-30499-x (PMC9122904; doi:10.1038/s41467-022-30499-x)
Supplement: Supplementary file 7 — Reporting Summary [file 41467_2022_30499_MOESM7_ESM.pdf]

nature portfolio

Corresponding author(s): Bin Wu, Yi Xue, and Shu Quan

Last updated by author(s): Apr 15, 2022

Reporting Summary

Nature Portfolio wishes to improve the reproducibility of the work that we publish. This form provides structure for consistency and transparency in reporting. For further information on Nature Portfolio policies, see our Editorial Policies and the Editorial Policy Checklist.

Statistics

For all statistical analyses, confirm that the following items are present in the figure legend, table legend, main text, or Methods section.

n/a	Confirmed	
☐	☒	The exact sample size (n) for each experimental group/condition, given as a discrete number and unit of measurement
☐	☒	A statement on whether measurements were taken from distinct samples or whether the same sample was measured repeatedly
☐	☒	The statistical test(s) used AND whether they are one- or two-sided<br>*Only common tests should be described solely by name; describe more complex techniques in the Methods section.*
☒	☐	A description of all covariates tested
☒	☐	A description of any assumptions or corrections, such as tests of normality and adjustment for multiple comparisons
☐	☒	A full description of the statistical parameters including central tendency (e.g. means) or other basic estimates (e.g. regression coefficient) AND variation (e.g. standard deviation) or associated estimates of uncertainty (e.g. confidence intervals)
☐	☒	For null hypothesis testing, the test statistic (e.g. F, t, r) with confidence intervals, effect sizes, degrees of freedom and P value noted<br>*Give P values as exact values whenever suitable.*
☒	☐	For Bayesian analysis, information on the choice of priors and Markov chain Monte Carlo settings
☒	☐	For hierarchical and complex designs, identification of the appropriate level for tests and full reporting of outcomes
☒	☐	Estimates of effect sizes (e.g. Cohen's d, Pearson's r), indicating how they were calculated

Our web collection on statistics for biologists contains articles on many of the points above.

Software and code

Policy information about availability of computer code

Data collection	We use Amber18 to collect MD simulation data.
Data analysis	SigmaPlot 12.0, Origin 8.6, Graphpad Prism 9.1.0, Pymol 2.1.1, Image J 1.52a, NMRFAM-SPARKY 1.2 powered by Sparky 3.115, NMRPipe9.6, python2.7, Amber18,OOPS 0.9, SCWRL 4.0, VMD 1.9.3

For manuscripts utilizing custom algorithms or software that are central to the research but not yet described in published literature, software must be made available to editors and reviewers. We strongly encourage code deposition in a community repository (e.g. GitHub). See the Nature Portfolio guidelines for submitting code & software for further information.

Data

Policy information about availability of data

All manuscripts must include a data availability statement. This statement should provide the following information, where applicable:

- Accession codes, unique identifiers, or web links for publicly available datasets
- A description of any restrictions on data availability
- For clinical datasets or third party data, please ensure that the statement adheres to our policy

The source data of main figures and supplementary figures are provided in the source data excel file. The backbone chemical shifts generated in this study have been deposited in the BioMagResBank under the accession code BMRB 51107 [https://bmrb.io/data_library/summary/index.php?bmrbId=51107] (1H, 15N and 13C backbone chemical shift assignment of Spy29-124), BMRB 51108 [https://bmrb.io/data_library/summary/index.php?bmrbId=51108] (1H, 15N and 13C backbone chemical shift assignment of Spy1-124) and BMRB 51109 [https://bmrb.io/data_library/summary/index.php?bmrbId=51109] (1H, 15N and 13C backbone chemical shift assignment of Spy29-124 in complex with Im7A3). Previously published structure used was obtained from the Protein Data Bank: 3O39 [https://www.rcsb.org/structure/3O39] (crystal structure of Spy). Source data are provided with this paper.

March 2021

Field-specific reporting

Please select the one below that is the best fit for your research. If you are not sure, read the appropriate sections before making your selection.

☒ Life sciences ☐ Behavioural & social sciences ☐ Ecological, evolutionary & environmental sciences

For a reference copy of the document with all sections, see nature.com/documents/nr-reporting-summary-flat.pdf

Life sciences study design

All studies must disclose on these points even when the disclosure is negative.

Sample size	No sample size calculations were conducted. Sample sizes were chosen based on Quan et al., eLife, 3:e01584, 2014, Huang et al., Nature, 537 (7619):202-206, 2016, and Zhang et al., Nat Chem Biol, 9(1):51-8, 2013.
Data exclusions	No data were excluded.
Replication	All experiments were repeated as indicated in the figure legends. The experimental findings were reliably reproduced.
Randomization	Randomization was not applied in this study. Samples were treated equally and were not grouped.
Blinding	Blinding was not applied in this study as the measurements are objective and not affected by investigators' bias.

Reporting for specific materials, systems and methods

We require information from authors about some types of materials, experimental systems and methods used in many studies. Here, indicate whether each material, system or method listed is relevant to your study. If you are not sure if a list item applies to your research, read the appropriate section before selecting a response.

Materials & experimental systems

n/a	Involved in the study	
☒	☐	Antibodies
☒	☐	Eukaryotic cell lines
☒	☐	Palaeontology and archaeology
☒	☐	Animals and other organisms
☒	☐	Human research participants
☒	☐	Clinical data
☒	☐	Dual use research of concern

Methods

n/a	Involved in the study	
☒	☐	ChIP-seq
☒	☐	Flow cytometry
☒	☐	MRI-based neuroimaging
